# Short-term memory and sentence comprehension in Catalan aphasia

**DOI:** 10.3389/fpsyg.2022.880398

**Published:** 2022-10-10

**Authors:** Io Salmons, Helena Muntané-Sánchez, Anna Gavarró

**Affiliations:** ^1^Acquisition and Pathology Lab, Departament de Filologia Catalana, Universitat Autònoma de Barcelona, Bellaterra, Spain; ^2^Centre de Linguïstica Teòrica, Universitat Autònoma de Barcelona, Barcelona, Spain

**Keywords:** aphasia, short-term memory, sentence comprehension, neuropsychological assessment, cognitive disorders, Catalan, syntactic comprehension

## Abstract

The main goal of the present study is to investigate visual and verbal short-term memory side to side with sentence comprehension in Catalan-speaking subjects with aphasia in comparison with subjects without brain damage. We aim to examine whether there are any significant correlations between their performance on short-term memory and comprehension tasks in order to evaluate the hypothesis that linguistic and memory deficits in aphasia are the result of a dysfunction of a common mechanism, usually short-term memory. Eigthy-four control subjects and twelve individuals suffering from different types of aphasia were assessed using the Catalan version of the *Comprehensive Aphasia Test* (CAT-CAT), which includes one recognition task and two digit and word span tests to evaluate visual and verbal short-term memory, respectively, as well as a sentence-to-picture comprehension task. The results showed that the performance of subjects with aphasia was significantly low on all tasks. Yet, the logistic regression analysis revealed that the magnitude of the differences between the control and experimental group varied across subtests, and that visual short-term memory was better preserved than verbal memory. The results also showed that there were no significant correlations between memory and language comprehension, which rules out the hypothesis that the deficits observed are due to a common underlying mechanism. Individual variation was also observed, specially on memory subtests, which suggest that memory impairments cannot explain the comprehension deficit in aphasia.

## 1. Introduction

The association between language and other cognitive skills in aphasia has been explored for long (see, among others, Archibald et al., 1967; Helm-Estabrooks et al., 1995; Kasselimis et al., 2013; Fonseca et al., 2016; Gonzalez et al., 2020). Despite the fact that aphasia has traditionally been considered a language-primary deficit (Geschwind, 1970), several studies have reported significant cognitive impairments in individuals with aphasia. In fact, there is evidence that cognitive abilities such as visual and auditory attention, verbal and visuospatial short-term and working memory or executive function are impaired in aphasia (Nicholas et al., 2005; Seniów et al., 2009; Murray, 2012; El Hachioui et al., 2014; Lee and Pyun, 2014; Choinski et al., 2020). For instance, in a review of 47 works on the subject, Fonseca et al. (2016) found that 61.3% of the studies reported a lower performance in nonverbal cognitive tasks of subjects with aphasia compared to healthy subjects. This has led several authors to question whether the core deficit in aphasia is linguistic in nature and to argue that the linguistic difficulties are rather caused by attention and/or memory dysfunctions (Murray, 1999; Aboitiz et al., 2006; McNeil et al., 2011; Code, 2018). However, this conclusion is controversial in view of many other studies reporting spared or relatively spared nonverbal cognition (Gathercole and Baddeley, 2001; Potagas et al., 2011; Murray, 2012; Fedorenko and Varley, 2016; Little et al., 2019).

The studies on the relationship between specific nonverbal and verbal cognitive skills in individuals with aphasia are also inconclusive. While some studies have reported significant associations between linguistic and nonverbal cognitive tasks (Fucetola et al., 2009; Murray, 2012; Lee and Pyun, 2014; Marinelli et al., 2017; Wall et al., 2017; Choinski et al., 2020), others have not found any significant relationship between them (van Mourik et al., 1992; Helm-Estabrooks, 2002; Ivanova et al., 2017; Little et al., 2019).

Also, most of the studies observed a great variability among individuals with aphasia in nonverbal cognitive measures which, as some have already argued (Murray, 2012; Marinelli et al., 2017), suggests that cognitive deficits alone cannot explain the linguistic impairments in aphasia. In fact, there are other factors that may explain the cognitive difficulties observed. Fonseca et al. (2016), who carried out a systematic review of studies published between 1995 and 2015, found that most of the subjects with post-stroke aphasia performed in several nonverbal cognitive tasks similar to left and right brain damaged subjects without aphasia. The authors interpreted these results as evidence against the assumption that aphasia, mostly caused by left hemisphere damage, causes nonverbal cognitive deficits, and argued instead that the nonverbal cognitive impairment may be the result of the brain dysfunction itself or even dementia, despite the fact that the relationship between aphasia and dementia is not well-understood yet. Other researchers like, El Hachioui et al. (2014) argued that depression could be the reason behind the cognitive deficits in individuals with aphasia, since they are much more prone to suffer from depression than healthy subjects.

Although several authors have pointed out that other variables could account for the lack of a consistent pattern in aphasic performance, there is discrepancy on the factors that interact with cognitive function. According to Helm-Estabrooks et al. (1995), age, educational level, and the severity of aphasia did not influence the aphasic performance in nonverbal cognitive tasks. By contrast, Marinelli et al. (2017) found that a higher educational level did correlate with a higher performance in the nonverbal tasks from the *Cognitive Test Battery for Global Aphasia* (Marinelli et al., 2006), but not age or time post onset. Murray (2012) argued that different patterns of performance could be associated with different aphasia types, as her findings showed that only individuals with anomic aphasia presented relatively spared attention measured through the *Test of Everyday Attention* (Robertson et al., 1994), whereas variables like age, education or aphasia severity did not have any effect on performance. Laures-Gore et al. (2011), on the other hand, found a significant association between severity of aphasia and working memory measures, as well as Choinski et al. (2020). In summary, even though there is wide evidence that individuals with aphasia tend to present nonverbal cognitive deficits, it is still unclear to what extent and what causes them, and whether they are directly correlated or not with their linguistic deficits.

To further investigate this issue, in the present study we examine visual and verbal short-term memory (STM), which refers to the ability to temporary store and maintain information (Baddeley, 2003), and its relationship to language skills. The relevance of memory in the linguistic deficits observed in aphasia was already pointed out in the first descriptions of aphasia (Benton and Joynt, 1960; Luzzatti and Whitaker, 1996). In more recent years, many authors (e.g., Baddeley, 2003; Aboitiz et al., 2006; Sung et al., 2009; Potagas et al., 2011) have claimed that individuals with aphasia present primary deficits in STM or working memory (WM), a specific form of STM that is responsible for the manipulation of the information while executing cognitive operations (Baddeley and Hitch, 1974). The Baddeley and Hitch's STM model involves different components, among which a phonological loop and a visuospatial sketchpad as temporary storage systems of verbal-acoustic and a visuospatial information. In addition, the central executive system was postulated as an attentional control system. According to Baddeley (2003), conduction aphasia is the paradigmatic example of a disruption of the verbal STM which results in the impaired ability to repeat sentences and sequences of digits.

STM deficits in fact have been observed in different types of aphasia. There is ample evidence that people with aphasia (PWA) often perform worse than control subjects on span tasks that evaluate WM and STM (e.g., Friedmann and Gvion, 2003; Seniów et al., 2009; Christensen and Wright, 2010; Laures-Gore et al., 2011; Wall et al., 2017; Christensen et al., 2018). Yet, as already discussed, the results are subject to great variability. Potagas et al. (2011), for example, conducted a study on spatial and verbal short-term and working memory of a group of PWA, whose poor performance on both types of memory correlated with the severity of the aphasia. They used a battery of tests that included the WAIS-III digits forward and backward tests, the Corsi block-tapping task and the Greek *Boston Aphasia Examination—Short Form* (Tsapkini et al., 2009). They also observed a dissociation between both types of memory in most patients, as spatial memory was intact in 43 out of 58 patients and, interestingly, the results between spatial and verbal memory did not correlate. The authors interpreted these findings as evidence against the assumption that there is an underlying common mechanism that explains language and memory deficits in aphasia, and argued that other variables would account for the covariance between memory and language deficits and aphasia severity.

Similarly, Lang and Quitz (2012) found that the results on memory tasks from the *Kurze Aphasieprüfung KAP Short Assessment for Aphasia* (Lang et al., 1999) and the *Wechsler Memory Scale Revised* (Härting et al., 2000) correlated with the severity of aphasia, rather than the type of aphasia. They also observed that verbal tasks were more likely to be impaired than nonverbal tasks, which they interpreted as evidence of disruption of a more general component of WM in contrast with Potagas et al. (2011). On the other hand, Fedorenko and Varley (2016) examined neuroimaging data from a group of subjects with severe global aphasia and concluded that they were able to carry out nonverbal cognitive tasks that required storage of information in working memory. Gathercole and Baddeley (2001) also claimed that nonverbal cognition is preserved in Broca's aphasia. Hence, despite some lack of consistency in the data, verbal STM seems to be more affected than nonverbal memory in aphasia.

In the present study, we also assess the auditory comprehension of sentences which requires, among others, syntactic and semantic knowledge, and investigate whether it significantly correlates with STM measures. In spite of the fact that anomia was traditionally considered the core deficit in aphasia, from the 70's sentence comprehension has been extensively investigated in several languages, and it is well-known that it is compromised even in non-fluent aphasias (e.g., Caramazza and Zurif, 1976; Schwartz et al., 1980; Grodzinsky, 1990; Hickok et al., 1993; Grillo, 2009). The comprehension deficit of certain sentence types has been explained in terms of memory impairment (e.g., Hickok, 2000; Stowe, 2000; Aboitiz et al., 2006; Varkanitsa and Caplan, 2018), and also as the result of linguistic deficits (e.g., Grodzinsky, 2000; Bastiaanse and van Zonneveld, 2005; Friedmann, 2006; see Garraffa and Fyndanis, 2020). In fact, both language and STM are argued to rely on extensive neural networks and have been often located in the same areas (see Varkanitsa and Caplan, 2018 for a review), in particular, phonological working memory relies on extensive temporoparietal-prefontal connections according to Hickok and Poeppel (2000) or the inferior frontal gyrus according to Aboitiz et al. (2006). According to Caplan and Waters (1999), the single-resource (SR) hypothesis makes the prediction that the accuracy of participants in a comprehension task is associated with their memory capacity, whereas the separate-sentence-interpretation-resource (SSIR) hypothesis makes the opposite prediction, that is, that memory capacity does not affect the accuracy of participants in comprehension tasks.

Many studies have found significant correlations between the performance of PWA on STM and sentence comprehension tasks (Fucetola et al., 2009; Seniów et al., 2009; Sung et al., 2009; Christensen and Wright, 2010; Lee and Pyun, 2014; Wall et al., 2017; Zakariás et al., 2018). Varkanitsa and Caplan (2018) conducted a meta-analysis of studies published between 1980 and 2017 and concluded that the results showed significant associations between STM and sentence comprehension. Some authors interpreted them as evidence that STM measures could predict the comprehension abilities of PWA, whereas others claimed that language difficulties explained the STM deficits (see Wright and Fergadiotis, 2012 for a review). Yet, these correlations could reflect the influence of other factors rather than causal relationships and, in any case, they do not reveal whether the common cause is a language- or a memory-related deficit. One of the challenges of assessing cognitive skills is that most behavioral tasks rely on different cognitive domains that cannot be tested separately. STM is required in sentence comprehension and, similarly, linguistic abilities are needed to complete verbal STM tasks.

In summary, despite many unsolved issues, it is well-established that aphasia often involves non-linguistic cognitive deficits that need to be assessed (and subsequently taken into consideration in treatment). Thus, further research is needed to examine the relationship between linguistic and other cognitive skills in aphasia. The goal of the present study is to investigate visual and verbal short-term memory as well as sentence comprehension of Catalan-speaking subjects with different types of aphasia and, in the second place, to examine whether there is any correlation between their performance on the short-term tasks and their scores in the sentence comprehension task in order to establish the nature of the deficit.

Let us consider the hypothesis that linguistic and memory deficits in aphasia are the result of a dysfunction of a common mechanism. If so, the prediction that follows is that PWA will perform poorly on all tasks, but also that a significant correlation between STM and language comprehension subtests will be observed. Moreover, an association between visual and verbal STM scores is to be expected if a common component of STM, namely the central executive (Baddeley and Hitch, 1974), is affected. On the other hand, if the memory deficit is domain-specific and affects only one of the two subsystems responsible for the storage of information, then a dissociation between the PWA's performance on visual and verbal tasks is predicted, as well as a significant correlation between the impaired STM type and the comprehension task [in line with the SR hypothesis postulated by Caplan and Waters (1999)].

Alternatively, if the primary impairment in aphasia is language-related but not dependent on memory capacity (following the SSIR hypothesis and other grammatical characterizations of aphasia), we would predict poor performance of PWA on comprehension and, to a lesser extent, verbal STM tasks, since linguistic skills are required to carry them out. Importantly, significant correlations between STM and comprehension subtests would not be expected, given that sentence comprehension and STM tasks rely mainly on different cognitive mechanisms. This does not exclude the possibility of more general cognitive impairments in some individuals, in line with previous research.

## 2. Materials and methods

### 2.1. Participants

Eighty-four control subjects of ages between 18 and 90 years (*M* = 50.6) participated in the study, as well as twelve participants of ages between 44 and 92 years (*M* = 58.5) suffering from different types of chronic aphasia, due to acquired brain injury in the left hemisphere. All the participants were bilingual speakers of Catalan and Spanish, though Catalan was their dominant language (and they spoke various dialects of Catalan), and they had different educational backgrounds (Table 1). According to a self-report, none of the control participants had a history of brain injury, cognitive disorders, or learning disabilities.

**Table 1:** Demographic characteristics of the participants.

	**Control group *n* = 84**	**Aphasic group *n* = 12**
**Age (years)**		
18–29	18 (21.4%)	0 (0%)
30–39	8 (9.5%)	0 (0%)
40–49	12 (14.3%)	5 (41.7%)
50–59	17 (20.2%)	3 (25%)
60–69	14 (16.2%)	1 (8.3%)
70–79	8 (9.5%)	2 (16.7%)
+80	7 (8.3%)	1 (8.3%)
**Education**		
Basic	18 (21.4%)	0 (0%)
Intermediate	34 (40.5%)	5 (41.7%)
Higher	32 (38.1%)	7 (58.3%)
**Sex**		
Female	50 (59.9%)	7 (58.3%)
Male	34 (40.5%)	5 (41.7%)
**Dialect**		
Western	30 (35.7%)	0 (0%)
Eastern	54 (64.3%)	12 (100%)

The participants with different types of aphasia were selected from the patient pools of several hospitals in the Barcelona area, namely the Hospital Sant Rafael, the Escola de Patologia del Llenguatge de l'Hospital de Sant Pau and the Grup de Suport Neuropsicològic. The inclusion criteria involved self-reported language and cognitive skills within normal range before the stroke. Details of age, education, handedness, etiology, aphasia subtype, and time post-onset collected from the medical health record of the PWA appear in Table 2. The participants with aphasia were diagnosed by speech pathologists and neurologists in the abovementioned centers according to clinical criteria and formal or informal tests mostly carried out in Spanish, since there are no available standardized tools in Catalan to diagnose aphasia. Since one of the goals of the study was to determine the feasibility of cognitive testing with CAT-CAT, the aphasic sample represents the variability found in clinical practice (The CAT does not aim at discriminating among aphasia types and does include an aphasia severity scale; Swinburn et al., 2004).

**Table 2:** Individual characteristics of subjects with aphasia.

**Subject**	**Age**	**Sex**	**Education**	**Handedness**	**Etiology**	**TPO (years)**	**Aphasia**
1	78	F	3	Right	Tumor	12	Anomic
2	56	F	3	Right	CVA	9	Anomic
3	92	M	2	Right	CVA	0.84	Conduction
4	47	F	3	Right	Tumor	5	Global
5	45	M	2	Right	CVA	9	TM
6	78	F	2	Right	HCVA	2	TM
7	44	M	3	Right	HCVA	3	TM
8	54	F	2	Right	CVA	10	Wernicke
9	48	M	2	Right	CVA	6	–
10	60	M	3	Right	CVA	8	–
11	49	F	3	right	HCVA	11	–
12	51	F	3	right	Tumor	24	–

Sex: F, female; M, male. Education: 1, basic; 2, intermediate; 3, higher. Etiology: CVA, cerebrovascular accident; H, hemorrhagic; TPO, time post-onset. Aphasia: TM, transcortical motor aphasia.

A *chi*-square goodness of fit test revealed that the proportions in both groups differed by dialect use (χ^2^ = 10.57, *p* = 0.005), but not by education level (χ^2^ = 5.76, *p* = 0.056). On the other hand, a Wilcoxon-Mann-Whitney and a Fisher exact tests indicated that there were no significant differences between the two groups with respect to age (*U* = 658.5, *p* = 0.399) and sex (two-tailed *p* = 1).

### 2.2. Materials

The participants were administered the Catalan version of the *Comprehensive Aphasia Test* (CAT; Swinburn et al., 2004) adapted by Salmons et al. (2021), which consists in a battery of twenty-seven subtests that evaluate both cognitive and linguistic skills, including the four behavioral tasks reported in the present study. Three of these tasks assessed visual and verbal short-term memory, and required the storage and maintenance of information (Baddeley, 2007). These type of tasks have been shown to be appropriate for use with subjects with brain damage (Wright and Fergadiotis, 2012). The last task evaluated the comprehension of different sentence types. The words and structures tested are not subject to variation between Eastern and Western Catalan, and therefore the tasks were suitable for all participants independently of the dialect they spoke.

#### 2.2.1. Visual short-term test

The first test assessed visual STM through a recognition task, and it included a practice item and ten experimental items. The participants were presented with four images and were asked to point to the one that they had already seen in a previous task, administered at least 3 min before, that evaluated semantic memory. Each token includes the target picture and three unrelated distractors; the pictures can be found in Salmons et al. (2021), and cannot be reproduced here since they are subject to copyright. The score was the number of correct responses.

#### 2.2.2. Verbal short-term memory tests

Verbal SMT was measured through two repetition tasks in which subjects were asked to repeat digit strings and sentences that gradually increased in length. The digit repetition test (or digit span test) consisted in a total of six tokens, starting with a two-digit string (e.g., *7 2*) and increasing the length by one in each token up to a string of seven digits (e.g., *2 8 7 4 5 1 2*). The score was the number of digits that the participant was able to repeat without mistakes (digit span). All numbers were monosyllables (*vuit* “eight”), except for number four (*qua.tre*).

In the sentence repetition task (or sentence listening span test), participants were asked to repeat sentences that gradually increased in length starting from sentences with three content words (1) to sentences with six content words. The complete list of the items can be found in the Supplementary materials.

(1) El gat atrapa el ratolí.
  “The cat catches the mouse.”

The score was the number of content words (e.g., nouns and verbs) that the participant was able to repeat without error (listening span). Function words are not taken into account in this type of task since the aim of the test is to evaluate the memory capacity rather than the grammatical competence of participants (Swinburn et al., 2004). As an anonymous reviewer pointed out, difficulties in the repetition of grammatical morphemes could possibly be associated with agrammatism, but the scoring system of CAT does not contemplate these measures given the goal of the test—see, however, the discussion on this point. In fact, other types of repetition tasks are used to evaluate the linguistic abilities of PWA (e.g., Friedmann, 2001) and other populations (see, for example, Lust et al., 1996; Marinis and Armon-Lotem, 2015).

In both tasks, each level had two trials. Hence, the participants had two opportunities per level to repeat the digit string or the sentence correctly. If the participant failed to repeat the digit string or the sentence on the first trial, the examiner asked him/her to repeat a second string or sentence in the same level. If the participant repeated it correctly, the examiner proceeded with the first trial of the next level. However, if the participant failed to repeat both trials at one level, the task was terminated. Phonetic and dyspraxic errors were not taken into account.

#### 2.2.3. Sentence comprehension test

**Figure 1:**
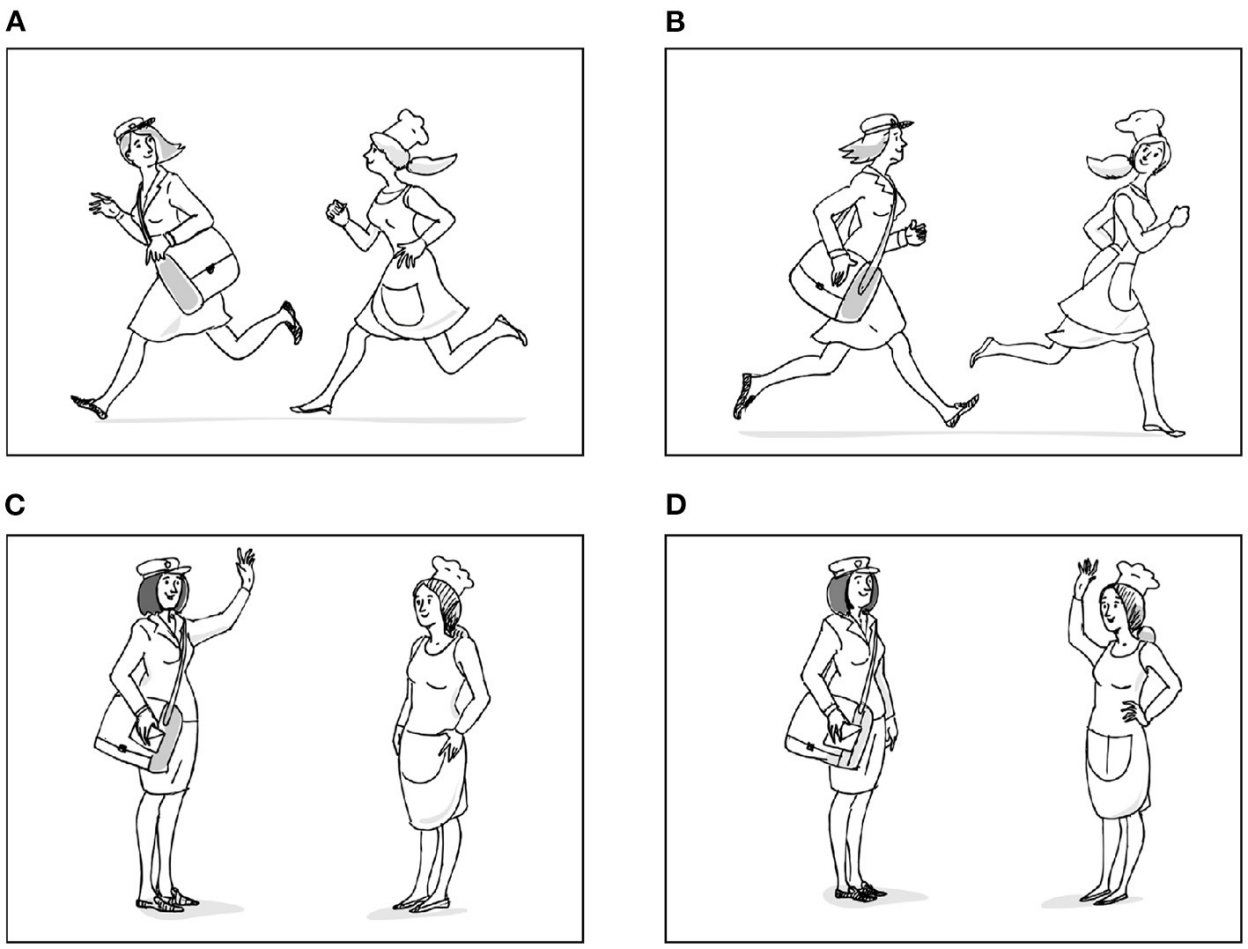
**(A–D)** Materials presented in the sentence comprehension task for the item in (2b). Reproduced with permission from Servei de Publicacions de la UAB, available at https://ddd.uab.cat/pub/llibres/2021/250143/prointafa_a2021b.pdf.

The fourth subtest consisted in a sentence-picture matching task. The participants were shown four pictures and were asked to listen to a sentence read by the examiner, and point to the picture that matched the sentence heard (Figure 1). The task included a total of 18 sentences with simple and common words (2), given that its purpose is to detect syntactic disorders rather than lexical difficulties (Swinburn et al., 2004). The main goal of the task is to identify a problem in sentence comprehension without going into the diagnosis of aphasia type, and so a wide range of syntactic structures are included, such as active sentences with one and two arguments (2a), or constructions that have been shown to be problematic in aphasia like passives and clitic left dislocations (2b) (see Gavarró and Dotti, 2014; Salmons, 2015 for Catalan).

(2)
  - L'home menja una poma.
    the-man eats an apple
    “The man is eating an apple.”
  - A la  cartera, la persegueix la cuinera.
    to the postwoman her chases   the cook
    “The postwoman, the cook is chasing her.”

The task includes five active sentences with two arguments, three active sentences with one argument, two passives, two clitic left dislocations, one sentence with a relative clause, and five sentences with Prepositional Phrases (PP). Whereas, all passives and clitic left dislocations were semantically reversible sentences, only some of the actives with two arguments and PPs were semantically reversible. The complete list of items can be found in the Supplementary materials.

The materials for semantically reversible sentences (2b) included the target picture, and two lexical foils and a role reversal as distractors (Figure 1). The agents were placed both on the left and on the right of the picture to control for any bias resulting from picture paralleling the order of mention of the subjects and objects. There were two scoring systems in this task. The first one is based on a 0–1 scale: correct responses scored 1, whereas incorrect or no responses scored 0. On the other hand, the second one is based on a 0–2 scale: incorrect or no responses scored 0; correct responses initiated within the first 5 s were assigned a value of 2; correct responses after a 5 s-delay, a repetition of the item on request or a self-correction were penalized and given a value of 1. By taking into account these variables, this latter system allows to assess qualitative differences in the participants' responses (Howard et al., 2010). An anonymous reviewer pointed out that the 5 s cuttoff of the *Comprehension Aphasia Test* may be too long as it exceeds by far a natural pause/delay, and may reflect difficulties with executive function (decision making), attention, memory, or motor function to execute the response; therefore one might have expected a shorter cuttoff point to evaluate the participant's delay in producing an answer. Admittedly, response time cutoffs vary from test to test (see Evans et al., 2020 for a review), but other aphasia diagnostic tools use the 5 s cutoff, for example the *Boston Diagnosis Aphasia Evaluation* (Goodglass et al., 2001) in its word comprehension task. Therefore, the choice made in the CAT is by no means exceptional and we adhere to it here.

### 2.3. Procedure

The procedure of the study was approved by the UAB Ethics Committee (CEEAH 5656) and followed the Declaration of Helsinki ethical standards. All the subjects provided their written informed consent prior to participation. Participants were tested in medical institutions or at home by trained examiners. The experimental tasks were conducted in a quiet room to minimize distractions. Control subjects completed the CAT-CAT (Salmons et al., 2021) in a 40 min session, whereas subjects with aphasia needed up to 1 h sessions. Prior to the task, the participants were interviewed to collect relevant personal and medical information. Then the examiner explained the task. All the tests included one practice item in order to ensure that the participants comprehended the task. Once the practice item was performed correctly, the experimental items were presented and the responses were collected manually.

### 2.4. Statistical analysis

Descriptive statistics were presented as mean, standard deviation, median, range and interquartile range. Distribution for normality was assessed with the Shapiro–Wilk test. The Mann–Whitney-Wilcoxon test was used to compare the distributions of the two groups given the non-normal distribution of the data. Since the sample size for the aphasic group was small, a fixed effects model for binomial data was performed to determine whether there was a significant effect of group, to model the probability of success, and to calculate the odds ratio and 95% confidence intervals. Cutoff values were also calculated for each subtest in order to discriminate impaired and unimpaired performance. The cutoff points corresponded to the score that the 95% of control participants exceeded, following the methodology used in other versions of the CAT (Swinburn et al., 2004; Zakariás and Lukács, 2021). Scores below the cutoff represent impaired performance. A logistics regression analysis was conducted to estimate and compare the percentages of participants at or above the cutoff point in both groups. Finally, Pearson and Spearman correlation coefficients were used to measure lineal and monotonic associations between the four tasks. The correlation coefficients were corrected using the Bonferroni correction method. Statistical significance was set at *p* < 0.05. The statistical analysis was performed using SAS software, version 9.4 (SAS Institute Inc., USA).

## 3. Results

The mean correct responses and other descriptive statistics of the control and experimental groups in the four tasks appear in Table 3. The individual scores of the PWA and cutoff points appear in Table 4, as well as results for other subtests that assessed relevant skills, namely, semantic memory, and word repetition and comprehension.

**Table 3:** Correct responses by task and group.

**Subtest**	**Max**	**Group**	**Mean**	**SD**	**Range**	**Median**	**IQR**
**Visual STM**	10	Control	9.69	1.15	2–10	10	10–10
		Aphasic	9.08	1.51	5–10	10	8.5–10
**Verbal STM**						
Digit span	7	Control	5.99	1.11	3–7	6	4–7
		Aphasic	4	1.13	2–6	4	3.5–4
Listening span	6	Control	5.99	0.11	5-6	6	6–6
		Aphasic	4.75	1.22	3–6	4.5	4-6
**Sentence comprehension**						
0–1 scale	18	Control	17.61	0.68	14–18	18	17–18
		Aphasic	13.67	2.71	7–17	14	12.5–17
0–2 scale	36	Control	34.39	1.87	24–36	35	24–35
		Aphasic	22.17	5.83	12–33	23.5	12–23.5

IQR, interquartile range; Max, maximum score; SD, standard deviation.

**Table 4:** Individual correct responses of subjects with aphasia by task.

**Subtest**	**Cutoff**	**Subjects with aphasia**											
		**1**	**2**	**3**	**4**	**5**	**6**	**7**	**8**	**9**	**10**	**11**	**12**
Arithmetic	5/6	4	4	1	5	3	1	5	5	5	4	6	6
Semantic memory	9/10	10	9	7	10	9	7	10	10	10	10	10	10
Visual STM	9/10	9	10	5	10	10	8	10	9	10	10	8	10
**Verbal STM**						
Digit span	4/7	6	4	3	4	4	6	3	4	4	2	4	4
Listening span	6/6	6	6	4	3	6	6	3	5	4	4	4	6
**Word repetition**						
0–1 scale	16/16	16	15	10	16	16	15	14	16	15	15	16	16
0–2 scale	31/32	32	30	20	32	32	28	22	32	30	29	31	32
**Word comprehension**						
0–1 scale	14/15	12	15	9	15	14	12	14	15	14	15	15	15
0–2 scale	28/30	24	30	17	29	25	20	27	29	27	30	28	30
**Sentence comprehension**						
0–1 scale	17/18	17	12	7	14	13	14	14	15	13	12	17	16
0–2 scale	32/36	33	19	12	27	24	26	16	22	23	15	24	25

### 3.1. Visual short-term memory

Both groups obtained high scores on the visual STM task, but the control participants' mean of correct responses (*M* = 9.69, *SD* = 1.15) was slightly higher than the aphasics' (*M* = 9.08, *SD* = 1.51). The responses of both groups were not normally distributed (*W*_*Control*_ = 0.29, *p* < 0.001; *W*_*Aphasic*_ = 0.68, *p* = 0.001), and their distributions differed significantly, *U* = 437, *p* = 0.016. The fixed-effects model revealed a significant main effect of group, *F* = 9.47, *p* = 0.003, and indicated that the estimated success rate for the control group was of 96.9% (95% CI [95.5, 97.9]), and of 90.8% (95% CI [84.1, 94.9]) for PWA. According to the parameters of the model, the probability of success of the control participants was three times higher than the aphasics' (*OR* = 3.26, 95% CI [1.5, 6.64]). This difference was statistically significant, *t* = 3.08, *p* = 0.003.

If we look at the individual responses as shown in Table 4, nine out of the twelve PWA scored at or above the cuttoff value. Two of the three participants who did not obtained a very close score of 8/10, while only one patient scored very low (5). However, the difference between both groups' percentages of participants that reached the cutoff point was significant (Fisher's exact test = 8.23, *p* = 0.025). In addition, the estimated percentages of passing the cutoff point of control and PWA was of 96.4% (95% CI [89.4, 98.9]) and 75% (95% CI [44.4, 91.9]), respectively. The difference was significant, *t* = 2.47, *p* = 0.015, the odds of the control group being nine times higher than the aphasics' (95% CI [1.54, 52.57]).

### 3.2. Verbal short-term memory

#### 3.2.1. Digit span

On average, the PWA obtained a lower digit span (*M* = 4, *SD* = 1.13) than the control subjects (*M* = 5.99, *SD* = 1.11). The normality test showed that the responses were not normally distributed (*W*_*Control*_ = 0.81, *p* < 0.001; *W*_*Aphasic*_ = 0.84, *p* = 0.025). The difference between the two groups' distributions was significant, *S* = 313.5, *p* < 0.001. And despite the fact that nine subjects with aphasia obtained a score at or above 4, the cutoff point, the percentage of individuals that exceeded it differed significantly from the controls' (Fisher's exact test = 14.91, *p* = 0.005). Also, the generalized linear model revealed that there was an effect of group if we considered the cutoff point, *F* = 7.57, *p* = 0.007, and that the estimated percentages of the aphasic and control groups of reaching it was of 75% (95% CI [44.4, 91.9]) and 98.8% (95% CI [91.8, 99.8]), respectively. The difference was significant (*t* = 2.75, *p* = 0.007); in fact, the control participants' probability of reaching the cutoff was twenty-eight times higher than the PWA's (*OR* = 27.7, 95% CI [2.52, 303.83]).

#### 3.2.2. Sentence listening span

The aphasic group obtained a mean listening span of 4.75 (*SD* = 1.22), which was lower than the control group's mean of 5.99 (*SD* = 0.11). The distribution of the data was not normal (*W*_*Control*_ = 0.09, *p* < 0.001; *W*_*Aphasic*_ = 0.82, *p* = 0.015), and differed between the groups, *S* = 291, *p* < 0.001. Moreover, less than half of the subjects with aphasia (5/12) reached the cutoff point in this subtest, which differed significantly from the percentage of control subjects that exceeded it (Fisher's exact test = 44.88, *p* < 0.001). According to the logistic regression analysis, there was a main effect of group (*F* = 16.69, *p* < 0.001) with control subjects being 116 times more likely to score at or above the cutoff point than the PWA (*t* = 4.09, *p* < 0.001; *OR* = 116.2, 95% CI [11.52, 1172]).

### 3.3. Sentence comprehension

The mean correct responses of the subjects with aphasia was lower than the controls': 13.67 (*SD* = 2.71) and 17.61 (*SD* = 0.68) on the 0–1 scale scoring and 22.17 (*SD* = 5.83) and 34.39 (*SD* = 1.87) on the 0–2 scale scoring, respectively. The differences between groups were significant: *S* = 114, *p* < 0.001 and *S* = 97, *p* < 0.001 for scores based on the 0–1 and the 0–2 scales, respectively. With regard to the scores based on the 0–1 scale, which only takes account of correct and incorrect responses, the results of the fixed effects model revealed a main effect of group, *F* = 16, *p* < 0.001. The estimated percentage of success of PWA was only of 50% (95% CI [24.1, 75.9]), much lower than that of control subjects, 96.4% (95% CI [89.4, 98.9]). The difference was significant, *t* = 4, *p* < 0.001, the odds of success of the control group being 27 times higher than the aphasics' (95% CI [5.26, 138.65]). In this case, only two PWA reached the cutoff threshold. The percentage of individuals who scored at or above the cutoff point was significantly lower than that of the control participants (Fisher's exact test = 52.04, *p* < 0.001). The fixed effects model revealed an effect of group (*F* = 24.59, *p* < 0.001), the estimated percentage of exceeding the cutoff being of 16.7% (95% CI [4.1, 48.2]) and 95.2% (95% CI [87.9, 98.2]) for subjects with and without aphasia, respectively. The difference was significant, *t* = 4.96, *p* < 0.001, and the odds of success of the control group was 100 times the odds of the experimental group (95% CI [15.82, 632.16]).

If we consider the scoring system based on the 0–2 scale, only one participant scored above the cutoff point, which differed significantly from the percentage of control participants that scored at or above the cutoff point (Fisher's exact test = 65.42, *p* < 0.001). As previously stated, this scoring system is more sensitive as it takes into account variables such as delayed response, request for repetition and self-correction. Not only a main effect of group was observed, *F* = 22.57, *p* < 0.001, but the magnitude of the difference between the control and aphasic group was greater than the one observed on the 0–1 scale: *OR* = 297, 95% CI [27.49, 3208.4]; *t* = 4.75, *p* < 0.001.

### 3.4. Correlations between subtests

**Figure 2:**
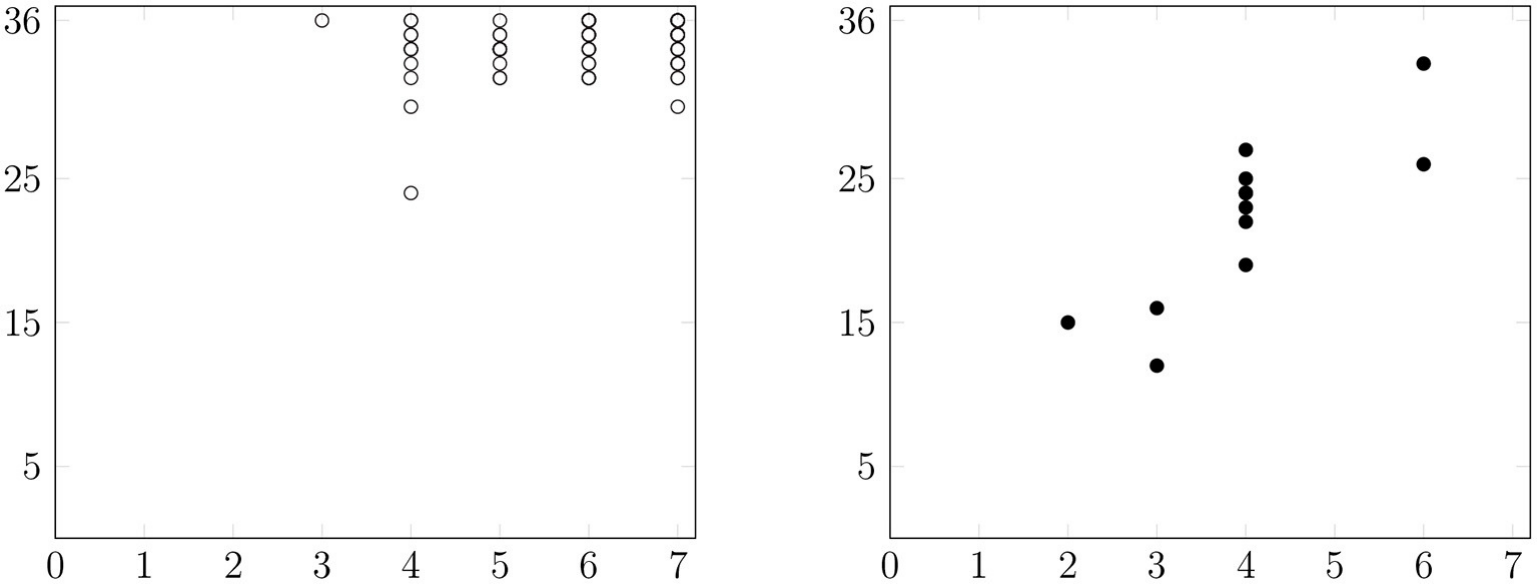
Relationship between the digit span (x) and scores based on the 0-2 scale on the comprehension task (y) of control subjects (open circles) and PWA (black-filled circles).

In order to establish the relationship between language comprehension and STM deficits in Catalan-speaking subjects with aphasia, correlations between the scorings on the four tasks were calculated (Tables 5, 6). Spearman correlation coefficients of 0.69 (*p* = 0.013) and 0.76 (*p* < 0.001) were obtained between the two scores of the comprehension subtest for aphasic and control subjects, respectively, indicating a moderate level of association. With regard to the performance of PWA, the score based on the 0–2 scale was strongly associated with their score on the digit span task [*r*_(10)_ = 0.85; *p* < 0.001; see Figure 2]. The results of the two verbal STM scores of the aphasic group were also moderately correlated, *r*(10) = 0.62; *p* = 0.03. After applying the Bonferroni correction test, the only significant association obtained was between the score of PWA based on the 0–2 scale in the comprehension task and digit span (Figure 2). Regarding control subjects, the only significant correlation observed was between the two scores of the comprehension subtest.

**Table 5:** Pearson correlation coefficients (subjects with aphasia).

	**Visual STM**	**Digit span**	**Listening span**	**Sentence comprehension**	

				**0–1 scale**	**0–2 scale**
**Visual STM**		−0.05	0.06	0.48	0.30
		*p* = 0.8687	*p* = 0.8479	*p* = 0.1179	*p* = 0.3454
		Adj *p* = 1.0000	Adj *p* = 1.0000	Adj *p* = 1.0000	Adj *p* = 1.0000
**Digit span**	−0.05		0.60	0.51	0.83
	*p* = 0.8687		*p* = 0.0405	*p* = 0.0934	*p* = 0.0008
	Adj *p* = 1.0000		Adj *p* = 0.4054	Adj *p* = 0.9336	Adj *p* = 0.0084
**Listening**	0.06	0.60		0.22	0.43
**span**	*p* = 0.8479	*p* = 0.0405		*p* = 0.4901	*p* = 0.1631
	Adj *p* = 1.0000	Adj *p* = 0.4054		Adj *p* = 1.0000	Adj *p* = 1.0000
**Sentence comprehension**					
0–1 scale	0.48	0.51	0.22		0.76
	*p* = 0.1179	*p* = 0.0934	*p* = 0.4901		*p* = 0.0038
	Adj *p* = 1.0000	Adj *p* = 0.9336	Adj *p* = 1.0000		Adj *p* = 0.0381
0–2 scale	0.30	0.83	0.43	0.76	
	*p* = 0.3454	*p* = 0.0008	*p* = 0.1631	*p* = 0.0038	
	Adj *p* = 1.0000	Adj *p* = 0.0084	Adj *p* = 1.0000	Adj *p* = 0.0381	

Adj, adjusted *p*-values (Bonferroni).

**Table 6:** Pearson correlation coefficients (control subjects).

	**Visual STM**	**Digit span**	**Listening span**	**Sentence comprehension**	

				**0–1 scale**	**0–2 scale**
**Visual STM**		0.02	0.07	−0.05	−0.02
		*p* = 0.8860	*p* = 0.5494	*p* = 0.6534	*p* = 0.8880
		Adj *p* = 1.0000	Adj *p* = 1.0000	Adj *p* = 1.0000	Adj *p* = 1.0000
**Digit span**	0.02		0.20	0.20	0.27
	*p* = 0.8860		*p* = 0.0724	*p* = 0.0662	*p* = 0.0116
	Adj *p* = 1.0000		Adj *p* = 0.7236	Adj *p* = 0.6616	Adj *p* = 0.1160
**Listening**	0.07	0.20		0.10	0.26
**span**	*p* = 0.5494	*p* = 0.0724		*p* = 0.3701	*p* = 0.0171
	Adj *p* = 1.0000	Adj *p* = 0.7236		Adj *p* = 1.0000	Adj *p* = 0.1714
**Sentence comprehension**					
0–1 scale	−0.05	0.20	0.10		0.85
	*p* = 0.6534	*p* = 0.0662	*p* = 0.3701		*p* < 0.001
	Adj *p* = 1.0000	Adj *p* = 0.6616	Adj *p* = 1.0000		Adj *p* < 0.001
0–2 scale	−0.02	0.27	0.26	0.85	
	*p* = 0.8880	*p* = 0.0116	*p* = 0.0171	*p* < 0.001	
	Adj *p* = 1.0000	Adj *p* = 0.1160	Adj *p* = 0.1714	Adj *p* < 0.001	

Adj, adjusted *p*-values (Bonferroni).

These results were in line with the Pearson correlation analysis (Tables 7, 8): both scores for the comprehension task were positively associated in the aphasic [*r*_(10)_ = 0.76; *p* = 0.004] and control [*r*_(82)_ = 0.85; *p* < 0.001] groups. Like before, the digit span of PWA was correlated with their score based on the 0–2 scale in the comprehension task [*r*_(10)_ = 0.83; *p* < 0.001; see Figure 2] and their listening span [*r*_(10)_ = 0.6; *p* = 0.04]. In addition, two weak associations were observed in the control group responses between their score based on the 0–2 scale in the comprehension task and their digit span [*r*_(82)_ = 0.27; *p* = 0.012], on the one hand, and their listening span [*r*_(82)_ = 0.26; *p* = 0.017] on the other. After applying the Bonferroni correction test to the Pearson correlation analysis, significant associations were observed between the two scores of the comprehension task in both groups, and between the score of PWA based on the 0–2 scale in the comprehension task and their digit span (Figure 2).

**Table 7:** Spearman correlation coefficients (subjects with aphasia).

	**Visual STM**	**Digit span**	**Listening span**	**Sentence comprehension**	

				0-1 scale	0-2 scale
**Visual STM**		−0.25	−0.10	−0.20	−0.03
		*p* = 0.4332	*p* = 0.7632	*p* = 0.5285	*p* = 0.9373
		Adj *p* = 1.0000	Adj *p* = 1.0000	Adj *p* = 1.0000	Adj *p* = 1.0000
**Digit span**	−0.25		0.62	0.56	0.85
	*p* = 0.4332		*p* = 0.0308	*p* = 0.0556	*p* = 0.0005
	Adj *p* = 1.0000		Adj *p* = 0.3078	Adj *p* = 0.5560	Adj *p* = 0.0050
**Listening**	−0.10	0.62		0.15	0.36
**span**	*p* = 0.7632	*p* = 0.0308		*p* = 0.6460	*p* = 0.2533
	Adj *p* = 1.0000	Adj *p* = 0.3078		Adj *p* = 1.0000	Adj *p* = 1.0000
**Sentence comprehension**					
0–1 scale	−0.20	0.56	0.15		0.69
	*p* = 0.5285	*p* = 0.0556	*p* = 0.6460		*p* = 0.0134
	Adj *p* = 1.0000	Adj *p* = 0.5560	Adj *p* = 1.0000		Adj *p* = 0.1339
0–2 scale	−0.03	0.85	0.36	0.69	
	*p* = 0.9373	*p* = 0.0005	*p* = 0.2533	*p* = 0.0134	
	Adj *p* = 1.0000	Adj *p* = 0.0050	Adj *p* = 1.0000	Adj *p* = 0.1339	

Adj, adjusted *p*-values (Bonferroni).

**Table 8:** Spearman correlation coefficients (control subjects).

	**Visual STM**	**Digit span**	**Listening span**	**Sentence comprehension**	

				**0–1 scale**	**0–2 scale**
**Visual STM**		0.10	0.26	0.10	0.10
		*p* = 0.3844	*p* = 0.0182	*p* = 0.3857	*p* = 0.3815
		Adj *p* = 1.0000	Adj *p* = 0.1824	Adj *p* = 1.0000	Adj *p* = 1.0000
**Digit span**	0.10		0.17	0.15	0.26
	*p* = 0.3844		*p* = 0.1215	*p* = 0.1684	*p* = 0.0166
	Adj *p* = 1.0000		Adj *p* = 1.0000	Adj *p* = 1.0000	Adj *p* = 0.1656
**Listening**	0.26	0.17		0.15	0.19
**span**	*p* = 0.0182	*p* = 0.1215		*p* = 0.1824	*p* = 0.0889
	Adj *p* = 0.1824	Adj *p* = 1.0000		Adj *p* = 1.0000	Adj *p* = 0.8891
**Sentence comprehension**					
0–1 scale	0.10	0.15	0.15		0.76
	*p* = 0.3857	*p* = 0.1215	*p* = 0.1824		*p* < 0.001
	Adj *p* = 1.0000	Adj *p* = 1.0000	Adj *p* = 1.0000		Adj *p* < 0.001
0–2 scale	0.10	0.26	0.19	0.76	
	*p* = 0.3815	*p* = 0.0166	*p* = 0.0889	*p* < 0.001	
	Adj *p* = 1.0000	Adj *p* = 0.1656	Adj *p* = 0.8891	Adj *p* < 0.001	

Adj, adjusted *p*-values (Bonferroni).

## 4. Discussion

One of the goals of the present study was to examine visual and verbal STM and sentence comprehension in Catalan aphasia. The aphasic performance was significantly worse than the performance of the control group on all tasks, but the magnitude of the difference varied remarkably across subtests. In addition, there were considerable individual differences among PWA, specially on STM tasks. We also investigated whether there were any significant correlations between STM and language comprehension tasks. The results showed that there were no significant correlations between STM and sentence comprehension tasks. In view of the results we argue that our findings are inconsistent with the assumption that the impairments observed in the four tasks are due to a single-system deficit, given that no significant associations were found between STM and comprehension measures.

### 4.1. Short-term memory deficits

Our results are in line with the literature reviewed above, according to which visual and verbal STM is often impaired in aphasia, but subject to individual variability (Gonzalez et al., 2020). Interestingly, the performance of PWA on the visual STM task was very high despite being significantly lower than the controls' and, as already pointed out, most participants reached the cutoff point. Other studies have already reported mild or no impairment in visuospatial STM (e.g., Seniów et al., 2009; Potagas et al., 2011).

In contrast, the results of the aphasic group on the verbal STM subtests were much lower and, if we look at the individual scores, fewer participants scored at or above the cutoff point on the sentence repetition task. In fact, the probability of PWA of scoring at or above the cutoff point decreased from 9 to 28 and 116 times less in visual, digit and sentence STM tasks, respectively.

The difference in performance between visual and verbal STM tasks has also been found in studies that assessed visuospatial and verbal STM through other measures (Seniów et al., 2009; Potagas et al., 2011). In our study, no significant correlations between visual and verbal STM subtests were found. These findings rule out the possibility that the deficit observed in STM tasks is due to a disruption of a common memory mechanism, more specifically the central executive, and rather suggest a selective impairment of the phonological loop. It could also be argued that the difficulties shown by the PWA in verbal span tasks are due to a deficit in repetition, a function that can be selectively impaired in aphasia (Geschwind, 1970). Yet, most of the subjects that participated in our study presented very high scores on a task that assessed their ability to repeat simple words (Table 4), and no significant correlations between the scores on this task and those on the STM tasks were observed. The only exception is subject 2, whose mistakes involved mainly low-frequency words (five out of six errors) and words with more than two syllables (four out of six errors).

The scores on the two verbal STM subtests did correlate significantly, which reflects the fact that both tasks evaluate the same subtype of memory but, as already mentioned, fewer PWA reached the cutoff threshold on the sentence repetition task than on the digit span subtest. Previous studies reported similar results (Martin and Ayala, 2004; Lang and Quitz, 2012). The possibility that the poor performance on the digit repetition task may be due to a problem with numerical skills can be ruled out based on the PWA's results on the arithmetic task (see Table 4). The lower performance on the listening span subtest could be attributed to the higher complexity of sentence repetition as a task, since it requires both production and comprehension skills. Based on the results on the word repetition task, the possibility of a word length effect (Baddeley, 2007) can be ruled out.

An anonymous reviewer pointed out that verbal spans may have reflected different types of production deficits, grammatical deficits in particular. We analyzed the sentences produced by PWA in this task, and found that PWA made a total of 28 errors involving content words: 24 omissions and 4 substitutions of content words. All the substitutions except one were synonyms or semantically related words (e.g., *llegir el conte* “to read the story” instead of *explicar el conte* “to tell the story”). In two cases, the error consisted in repeating the sentence in a different word order. Except for one case in which the participant produced a sentence with an infinitive as the main verb, as well as unfinished sentences (*El gat..*. “The cat...” instead of *El gat atrapa el ratol*í “The cat catches the mouse”), the resulting utterances were grammatical (e.g., *La dona tanca la porta* “The woman closes the door” instead of *La dona marxa i tanca la porta* “The woman leaves and closes the door”). The PWA omitted and substituted 17 and 9 grammatical morphemes, respectively, and one patient produced one extra preposition, but these omissions and substitutions were ancillary of the changes in content words, since they did not result in ungrammaticality. Therefore, even if function words were not taken into consideration for the purpose of evaluating short-term memory, it does not seem that the 12 subjects with aphasia that participated in the study produced ill-formed sentences in this particular task. Finally, most mistakes affected longer sentences, which suggests problems with PWA's memory capacity rather than agrammatism or difficulties with syntactically complex sentences, as Caplan and Waters (1999) argued.

### 4.2. Sentence comprehension deficits

The aphasic performance on the sentence comprehension task was significantly worse than the controls', which is in line with the results on the other versions of the CAT (see Table 9) and the other studies reviewed above. This was actually the task where the least participants scored at or above the cutoff point, this being 100 times less likely for PWA than for controls. In fact, their performance was more consistent than the results on the other tasks despite the heterogeneity of the sample. The deficit observed in this subtest cannot be due to an impairment in word comprehension (Baddeley, 2003), as the results of the aphasic group on a word-to-picture matching task revealed (Table 4). Also, no significant associations were found between word and sentence comprehension.

**Table 9:** Mean scores by subjects with aphasia in other versions of the CAT.

**Subtest**	**Max**	**Language**	***n***	**Mean**
Visual STM	10	Arabic (Abou El-Ella et al., 2013)	100	7.57
		Croatian (Kuvač Kraljevića et al., 2020)	114	8.82
		English (Swinburn et al., 2004)	194	8.81
		Hungarian (Zakariás and Lukács, 2021)	99	8.24
		Turkish (Maviş et al., 2021)	20	8.29
Digit span	7	Arabic (Abou El-Ella et al., 2013)	100	3.86
		Croatian (Kuvač Kraljevića et al., 2020)	114	4.14
		English (Swinburn et al., 2004)	195	3.94
		Hungarian (Zakariás and Lukács, 2021)	90	3.35
		Turkish (Maviş et al., 2021)	20	4.8
Listening span	6	Arabic (Abou El-Ella et al., 2013)	100	4.68
		Croatian (Kuvač Kraljevića et al., 2020)	114	3.5
		English (Swinburn et al., 2004)	195	3.21
		Hungarian (Zakariás and Lukács, 2021)	87	3.39
		Turkish (Maviş et al., 2021)	20	3.3
Sentece comprehension	32	Arabic (Abou El-Ella et al., 2013)	100	22.39
		Croatian (Kuvač Kraljevića et al., 2020)	114	21.15
		English (Swinburn et al., 2004)	195	18.73
		Hungarian (Zakariás and Lukács, 2021)	95	19.92
		Turkish (Maviş et al., 2021)	20	16.35

Max, maximum score; *n*, number of subjects.

The sentence comprehension task of the CAT does not aim at evaluating the comprehension of specific syntactic structures (Swinburn et al., 2004), yet it includes a wide range of constructions. If we look at the error distribution, the PWA made a total of 52 mistakes (see Table 10) that mainly involved semantically reversible sentences with non-canonical word orders, like passives or clitic left dislocations (2b), and with PPs such as *The glass is under the plate* which corresponded to the 34.6 and 36.5% of the total errors, respectively. On the other hand, fewer mistakes were found with irreversible (2a) and reversible canonical sentences (*The postwoman is chasing the cook*), which only represented 5.8 and 19.2% of all errors, respectively, or on irreversible object relatives (3.8%). These findings suggest that the problems to understand the sentences arose when their interpretation relied on particular syntactic operations. There is actually extensive crosslinguistic evidence that PWA present impairments in comprehension that affect specific syntactic structures like passives, object relatives or clitic left dislocations (among others, Caramazza and Zurif, 1976; Hickok et al., 1993; Grodzinsky, 2000; Luzzatti et al., 2001; Friedmann, 2008; Salmons and Gavarró, 2013; Varlokosta et al., 2014; Adelt et al., 2017; Terzi and Nanousi, 2018; Garraffa and Fyndanis, 2020). Moreover, the fact that the comprehension task of the CAT, despite being a general comprehension task, is sensitive to the specific linguistic deficits suggests that it is an efficient diagnostic tool for aphasia.

**Table 10:** Errors by sentence type in the comprehension subtest (subjects with aphasia).

**Sentence type**	**Errors/Items**	**%**
Actives with one argument	0/36	0
Actives with two arguments	13/60	21.7
Clitic left dislocations	7/24	29.2
Passives	11/24	45.8
Relative clauses	2/12	16.7
Sentences with PPs	19/60	31.7

We also took into consideration the results on the comprehension task based on the two scoring systems which, as expected, were correlated. The scoring system based on the 0–2 scale provides additional information as it penalizes response delays, repetition requests and self-corrections, and the individual scores were low. These low scores probably reflected the greater complexity of the task, given that it demanded not only sentence comprehension, but also spatial search, scene analysis, word retrieval and sentence-to-picture matching (Grodzinsky, 2010; Caplan et al., 2013). The cutoff point based on the controls' scores based on the 0–2 scale favors this view, since their results in sentence comprehension were very poor compared to the other tasks (Table 4). Interestingly, Sung et al. (2018) carried out a study on the production abilities of a group of Korean-speaking aphasics, and the results showed that task type related to individual WM capacity, but not to sentence type (active vs. passive).

### 4.3. The relationship between short-term memory and sentence comprehension

The present study also aimed at testing the hypothesis that the difficulties of PWA in STM and sentence comprehension tasks are due to a common cause, whether it be an attention (McNeil et al., 2011), memory (Caplan and Waters, 1999; Aboitiz et al., 2006) or language deficit (Christensen and Wright, 2010). If that was the case, we would expect strong correlations between STM and comprehension subtests. However, the observation that no significant associations were found between the scores on these subtests rules out this possibility and suggests that a single-system deficit cannot explain the impairments observed in these tasks. Rather, we hypothesize that the disrupted tasks reflect deficits in different cognitive systems, namely, short-term memory and language.

Other studies have reported significant associations between forward digit and listening spans and sentence comprehension tasks (among others, Sung et al., 2009; Lee and Pyun, 2014; Choinski et al., 2020), interpreted as evidence that a common underlying mechanism was affected, usually a component of the STM (see Wright and Fergadiotis, 2012 for a review). This view also finds support from the fact that short-term memory is needed in sentence comprehension and, hence, that a deficit in this domain probably impacts on comprehension (Baddeley, 2003). Under this view, it could be argued that the impairments observed in sentence comprehension were triggered by a memory disorder, given that sentences with syntactic dependencies require greater memory and impose higher processing demands (Caplan and Waters, 1999). However, this cannot account for the results from behavioral studies on the comprehension of other types of syntactic dependencies that, despite requiring STM, are well-comprehended by PWA (Santi and Grodzinsky, 2007).

Hence, even if memory and language measures correlated, a STM disorder could not explain the pattern of syntactic deficits observed in the comprehension tasks in the present study and in previous literature (see Grodzinsky, 2000 for a review).

While admittedly it is difficult to assess different cognitive systems in isolation and to identify the impact of memory in sentence comprehension tasks, we believe that in the present study the impact of STM disorders was better captured by the scoring system based on the 0–2 scale. In fact, significant correlations were found between the score on the sentence comprehension task based on the 0–2 scale and the digit and listening spans. Hence, even though higher spans did not translate into higher accuracy rates, the 0–2 scoring system reflected greater difficulties with the comprehension task correlating with lower spans (Figure 2). This, however, does not hold for all participants (e.g., 1, 7, and 10) which, in turn, highlights the variability of STM deficits in aphasia. As we have argued before, we believe this is due to the higher task demands of the comprehension subtest. In fact, some of the studies that have reported significant correlations between memory and sentence comprehension used tasks that could have been more sensitive to STM deficits. This is the case of studies that used complex span tasks to assess WM, which are cognitively more demanding (Ivanova et al., 2017), or sentence comprehension tasks that involved items that gradually increased in length requiring a greater memory load and, hence, relied heavily on short-term memory. To illustrate, Choinski et al. (2020) used both the Token Test (De Renzi and Faglioni, 1978) and a sentence comprehension task with 30 PWA, and they obtained lower scores in the first task. These findings are in line with Caplan and Waters (1999)'s hypothesis, since the scores based on the 0–2 scale could actually reflect post-interpretive processes, such as remembering and using the meaning of the sentence to perform the task of analyzing the images and pointing to the correct one. According to these authors, a contrast between an active and a passive sentences involves mainly interpretive processes, while contrasts like the ones included in the Token Test rather reflect post-interpretive processing.

Our findings are therefore compatible with the assumption that the primary deficit in aphasia is linguistic; this in itself does not exclude the presence of other cognitive impairments or less direct associations between STM and sentence comprehension (Caplan and Waters, 1999; Varkanitsa and Caplan, 2018), in line with the SSIR hypothesis. This would account for the fact that, despite the heterogeneity of the sample, the deficits observed in sentence comprehension were more consistent than the ones observed in STM tasks. In fact, as argued before, there is wide evidence that the pattern of performance of aphasics in sentence comprehension tasks is robust across languages (Drai and Grodzinsky, 2006a,b). In contrast, the deficits in STM tasks were subject to individual variability as several participants performed within the normal range, specially on the visual and digit span tasks (e.g., participants 1, 3, 6, and 10). Other studies have also observed dissociations among participants in non-linguistic cognitive measures (Seniów et al., 2009; Potagas et al., 2011; Caplan et al., 2013; Lee and Pyun, 2014; Gonzalez et al., 2020).

In our view, the conflicting results reported in some of the literature, as well as the discrepancies with regard to the correlation between memory and comprehension measures in aphasia, favor the idea that aphasia does not always entail memory disorders, and that memory deficits alone cannot explain the linguistic impairment, as others have already argued (Murray, 2012; Fonseca et al., 2016; Marinelli et al., 2017). The non-linguistic cognitive disorders that often co-occur with aphasia could be the result of different factors that have already been pointed out in the literature, including the presence of brain damage (Wright and Fergadiotis, 2012) and other medical conditions like dementia or depression (El Hachioui et al., 2014). This is consistent with the literature on cognitive abilities that states that brain-damaged patients present different deficits regardless of the hemisphere damaged or the presence of aphasia (see Fonseca et al., 2016 for a review), as well as with the observation that aphasia severity is often related to the STM capacity (Gonzalez et al., 2020) as more severe aphasias often involve larger lesions.

One of the arguments used to argue that a short-term memory impairment underlies the comprehension deficits observed in aphasia is that both functions have been located in the same brain areas (Hickok and Poeppel, 2000; Aboitiz et al., 2006; Choinski et al., 2020), including the inferior frontal gyrus (IFG). Neuroanatomical data from aphasic and healthy subjects support the co-occurrence of STM and comprehension disorders in aphasia. However, as argued by Santi and Grodzinsky (2007), these deficits reflect the multimodal nature of the brain damaged areas rather than a working memory deficit. Also, general cognitive impairments have been claimed to explain linguistic deficits in several clinical conditions such as schizophrenia (see Kuperberg, 2010 for a review), Down syndrome (Baddeley and Jarrold, 2007) or primary progressive aphasia (Gorno-Tempini et al., 2004). Yet, the relationship between linguistic and other cognitive abilities may vary across clinical populations. Little et al. (2019), for example, compared the performance on verbal and nonverbal cognition tasks of two groups of twenty subjects with aphasia and thirty subjects with schizophrenia. The results showed that while both groups presented significant linguistic deficits, a significant nonverbal cognitive impairment only occurred in schizophrenia. An association between verbal and nonverbal cognition tests was only observed in the schizophrenia group, in line with previous studies (Kerns, 2007). This also implies that, while there is clearly an association between verbal and nonverbal cognitive skills, it is not a direct one.

Taken together this lead us to the conclusion that non-linguistic short-term memory disorders often coexist with language deficits in aphasia. Yet, the present study and previous research show that non-linguistic cognitive deficits are subject to wide individual variability while linguistic deficits remain constant. This emphasizes the need to assess people with aphasia exhaustively in order to precisely describe each patient's profile and plan an adequate intervention program (see van Mourik et al., 1992; Hinckley et al., 2001; Helm-Estabrooks, 2002; Seniów et al., 2009; Murray, 2012; Kuzmina and Weekes, 2017; Marinelli et al., 2017; Fonseca et al., 2018; Zakariás et al., 2018).

There are some standardized tests in different languages that include both verbal and nonverbal cognitive tasks and are specifically designed to evaluate individuals with aphasia, such as the *Cognitive Linguistic Quick Test* (Helm-Estabrooks, 2001) or the *Comprehensive Aphasia Test* (Swinburn et al., 2004) in English. The CAT is one of the most used tools for that purpose (Salis et al., 2018), as it aims at evaluating both verbal and nonverbal cognitive skills in a relatively short administration time, which makes it suitable for clinical use. In the present study we presented four subtests of the Catalan version of the test (Salmons et al., 2021) that evaluate visual and verbal STM, and sentence comprehension, and we have shown that it can be a useful tool to evaluate Catalan-speaking subjects with aphasia. Yet, further research is needed to enlarge the PWA sample of the present study. Also, this study does not allow us to investigate whether different aphasia types correlate with different patterns of comprehension and STM impairment, given that the CAT does not distinguish among aphasia types; furthermore we were lacking in information about the participants in our sample. Similarly, the CAT is a tool developed to be used in clinical practice, and the sentence comprehension task does not allow to investigate whether there are significant correlations between the comprehension of different syntactic structures and STM. Therefore, establishing links, on the one hand, between aphasia types, and comprehension and STM and, on the other hand, between syntactic structures and STM deficits, remains a topic for future research.

## 5. Conclusion

The aim of the study was to investigate visual and verbal short-term memory and sentence comprehension in Catalan-speaking subjects with aphasia and to compare them with subjects without brain damage. Within the limits of our sample, the results showed that the aphasic performance was significantly worse than the controls' on the four tasks and, hence, that subjects with aphasia had difficulties in short-term memory and comprehension. Yet, the logistic regression analysis revealed that the magnitude of the differences between groups varied remarkably across subtests, and that visual short-term memory was much better preserved that verbal short-term memory. Moreover, our results showed that there were no significant correlations between short-term memory and language comprehension, which rules out the hypothesis that the deficits observed are due to a common underlying mechanism. We argued that the deficit in aphasia is linguistic in nature, though short-term memory disorders can also occur in some individuals. In fact, there were great individual differences among participants with aphasia, which emphasize the importance of a comprehensive assessment and, hence, the use of diagnostic tools like the CAT in clinical practice.

## Data availability statement

The datasets presented in this study can be found in online repositories. The names of the repository/repositories and accession number(s) can be found at: https://doi.org/10.5565/ddd.uab.cat/255717.

## Ethics statement

The studies involving human participants were reviewed and approved by UAB Ethics Committee (CEEAH 5656). The patients/participants provided their written informed consent to participate in this study. Written informed consent was obtained from the individual(s) for the publication of any potentially identifiable images or data included in this article.

## Author contributions

AG and IS contributed to the conception and design of the study. HM-S and IS organized the database. IS wrote the first draft of the manuscript. All authors contributed to manuscript revision, read, and approved the submitted version.

## Conflict of interest

The authors declare that the research was conducted in the absence of any commercial or financial relationships that could be construed as a potential conflict of interest.

## Publisher's note

All claims expressed in this article are solely those of the authors and do not necessarily represent those of their affiliated organizations, or those of the publisher, the editors and the reviewers. Any product that may be evaluated in this article, or claim that may be made by its manufacturer, is not guaranteed or endorsed by the publisher.
