# Supplementary material for: Short-term memory and sentence comprehension in Catalan aphasia
Source: Front Psychol. 2022 Oct 10;13:880398. doi: 10.3389/fpsyg.2022.880398 (PMC9590377; doi:10.3389/fpsyg.2022.880398)
Supplement: Supplementary file 1 [file Data_Sheet_1.pdf]

Supplementary Material

1 MATERIALS

The items included in the sentence repetition and sentence comprehension tasks in the Catalan version of the *Comprehensive Aphasia Test* (Salmons et al., 2021) are reproduced below.

1.1 Repetition of sentences

1. Three content words:
   a. El gat atrapa el ratolí.
      'The cat catches the mouse.'
   b. La nena menja una poma.
      'The girl eats an apple.'
2. Four content words:
   a. La dona marxa i tanca la porta.
      'The woman leaves and closes the door.'
   b. Han decidit pintar el menjador de blau.
      'They decided to paint the dining room blue.'
3. Five content words:
   a. Els nens escoltaven mentre el professor explicava el conte.
      'The children listened while the teacher read the story.'
   b. El mapa urbà és petit i difícil de veure.
      'The city map is small and difficult to see.'
4. Six content words:
   a. El nen i la nena pugen al cim i admiren les vistes.
      'The boy and the girl climb the hill and admire the view.'
   b. Va passar molt temps abans que la zona es considerés segura.
      'It was a long time before the area was pronounced safe.'

1.2 Comprehension of spoken sentences

1. L'home menja una poma.
   'The man is eating an apple.'
2. La cuinera saluda la cartera.
   'The female cook is saying hi to the postwoman.'
3. Ella riu.
   'She is laughing.'
4. La cartera és perseguida per la cuinera.
   'The postwoman is being chased by the female cook.'
5. El gos seu damunt la taula.
   'The dog is sitting on the table.
6. La catifa on hi ha la gata és grisa.
   'The carpet the cat is on is red.'

7. El pallasso empeny el bomber.
   ‘The clown is pushing the firefighter.’
8. El got sota el plat és blau.
   ‘The glass under the dish is blue.’
9. L’home camina.
   ‘The man is walking.’
10. La cuinera persegueix la cartera.
    ‘The female cook is chasing the postwoman.’
11. El bomber és fotografiat pel pallasso.
    ‘The firefighter is being photographed by the clown.’
12. La dona beu.
    ‘The woman is drinking.’
13. El plat vermell és sota el got.
    ‘The red dish is under the glass.’
14. La dona pinta la paret.
    ‘The woman is painting the wall.’
15. La flor dins la tassa és blava.
    ‘The flower in the cup is blue.’
16. El got és sota el plat.
    ‘The glass is under the dish.
17. A la cartera, la persegueix la cuinera.
    ‘The postwoman, the female cook is chasing her.
18. Al bomber, el fotografia el pallasso.
    ‘The firefighter, the clown is taking a picture of him.’

REFERENCES

Salmons, I., Rofes, A., and Gavarró, A. (2021). *Prova integral d’afàsia. Llibre d’ítems* (Bellaterra: Servei de Publicacions de la UAB)
